# Severe Immune-Related Pneumonitis With PD-1 Inhibitor After Progression on Previous PD-L1 Inhibitor in Small Cell Lung Cancer: A Case Report and Review of the Literature

**DOI:** 10.3389/fonc.2019.01437

**Published:** 2019-12-18

**Authors:** Xiuju Liang, Yaping Guan, Bicheng Zhang, Jing Liang, Baocheng Wang, Yan Li, Jun Wang

**Affiliations:** ^1^Department of Oncology, No. 960 Hospital, The People's Liberation Army, Jinan, China; ^2^Department of Respiratory Medicine, Shandong Thoracic Diseases Hospital, Jinan, China; ^3^Cancer Center, Renmin Hospital, Wuhan University, Wuhan, China; ^4^Department of Oncology, The First Affiliated Hospital of Shandong First Medical University, Jinan, China; ^5^Shandong Provincial Qianfoshan Hospital, Shandong University, Jinan, China

**Keywords:** immune-related adverse event, programmed cell death 1 inhibitor, programmed cell death ligand 1, pneumonitis, immune checkpoint inhibitor

## Abstract

**Objective:** Combination therapy with programmed cell death protein-1 (PD-1) and programmed cell death ligand-1 (PD-L1) inhibitors might be viewed as a promising therapeutic strategy for resistant lung cancer, and it is becoming common that a second PD-1/PD-L1 inhibitor might be used following progression on previous PD-1/PD-L1 inhibitor. However, a subgroup of patients will experience various autoimmune toxicities, termed as immune-related adverse events (irAEs), that occur as a result of on-target and off-tumor inflammation.

**Materials and Methods:** In this report, we presented a patient with small cell lung cancer who received different PD-1/PD-L1 inhibitors during the course of disease progression. This patient experienced radiation-related pneumonitis, immune-related pneumonitis, as well as concomitant bacterial pneumonia.

**Results:** In particular, this patient developed immune-related pneumonitis with a second PD-1 inhibitor when she had a progressive disease on previous PD-L1 inhibitor. This patient was initially responsive to steroid treatment, but rapidly develop more severe pneumonitis and concomitant bacterial pneumonia with no response to antibiotics and steroid treatment. Finally, this patient got a good clinical response when receiving additional immunosuppressive medications infliximab and mycophenolate mofetil.

**Conclusions:** Patients with a history of radiation-induced pneumonitis and treated with sequential different PD-1/PD-L1 inhibitors have a relative high risk to develop high-grade or steroid-resistant pneumonitis, and additional immunosuppressive medications should be used earlier when severe pulmonary toxicity occurs.

## Introduction

Immune checkpoint inhibitors works by disrupting the PD-1 and PD-L1 direct interactions in the tumor microenvironment (1, 2). In clinical practice, anti-PD-1/PD-L1 antibodies have resulted in durable tumor remission and changed the treatment landscape in a variety of advanced cancers including small cell lung cancer (SCLC) and non-small cell lung cancer (NSCLC). Several PD-1 inhibitors (nivolumab, pembrolizumab, and avelumab) and PD-L1 inhibitors (atezolizumab and durvalumab) have been approved by US Food and Drug Administration (FDA) for treating multiple human solid tumors, based on improvements in survival outcomes.

**Figure 1:**
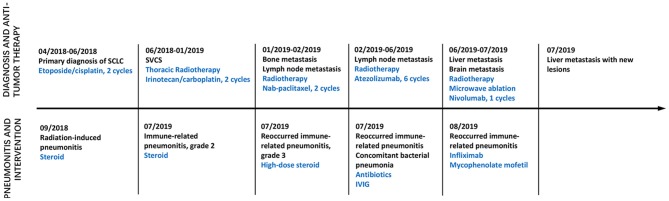
Time axis of anti-tumor treatment and intervention on pneumonitis. Line graph illustrating disease progression, anti-tumor therapy, pneumonitis and intervention from April 2018 to August 2019. IVIG, Intravenous immunoglobulin; SVCS, Superior vena cava obstruction syndrome.

Unlike cytotoxic chemotherapy, PD-1/PD-L1 inhibitors are usually manifested as tolerable agents, but 10–15% patients will develop grade 3–5 toxicity in non-target organs known as immune-related adverse events (irAEs) (3). Of these irAEs, pulmonary toxicity is one of the most dangerous side effects of PD-1/PD-L1 inhibitors, with a frequency of 5–10% in patients with lung cancer (4, 5). Pneumonitis associated with immunotherapy are generally uncommon but potentially fatal or life-threating (6). Generally, pneumonitis is more frequent in patients treated with anti-PD-1/PD-L1 antibodies compared to anti-CTLA-4 antibodies (7, 8), and more PD-1/PD-L1 inhibitor-related pneumonitis is observed in patients with lung cancer than those with melanoma (8). At present, combination therapy with PD-1/D-L1 inhibitors and other therapies is developing as a promising therapeutic strategy for advanced or metastatic lung cancer, and it is also becoming common that a second PD-1/PD-L1 inhibitor might be used following the disease progression on previous PD-1/PD-L1 inhibitor (9). These therapeutic strategies increase the frequency of an occurrence of irAEs including pneumonitis. Patients with pneumonitis related to PD-1/PD-L1 inhibitors may present clinically with drug cough, dyspnea, fever and chest pain, but radiologic findings often are non-specific (4, 5). Published guidelines or consensus can help clinically diagnose and manage irAEs, but general recommendations procedures are insufficient to resolve or relieve severe or complexed pulmonary toxicity (10–12). Here, we report a case with severe and rapidly developed reoccurred pneumonitis that occurred in the course of sequential use of PD-L1/PD-L1 inhibitors (Figure 1).

## Case Presentation

**Figure 2:**
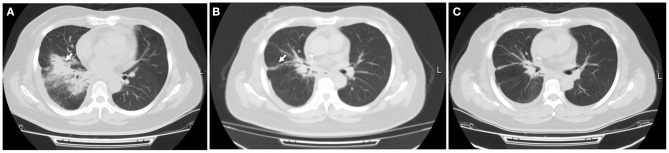
Radiation-induced pneumonitis. **(A)** After receiving three-dimensional thoracic radiotherapy, a CT scan showed new patchy opacity developed in the irradiated area of hilum of the right lung, which did not occur outside the irradiated area. **(B)** This patient initiated steroid treatment, pneumonitis was partially resolved within 1 month, with a significantly clinical improvement. **(C)** Pulmonary inflammation disappeared 2 months later. White arrow indicates inflammatory lesions.

In April 2018, a 44 year old woman was admitted to our hospital. She was initially diagnosed with localized SCLC (T2N1M0) through fiberoptic bronchoscopy in a local hospital. She did not experience other causes of obstructive lung disease, autoimmune disease, organ transplant, smoke inhalation, or medications. Molecular mutation analysis showed that the tumor did not harbor any driver gene alterations. Immunohistochemical staining of tumor tissue showed that PD-L1 expression was found in <1% of tumor cells. The tumor was located in right hilum of the right lung with multiple mediastinum lymph node metastasis. This patient received 2 cycles of first-line chemotherapy of etoposide (100 mg/m^2^ days 1–3, every 3 weeks) and cisplatin (100 mg/m^2^ every 3 weeks). Unfortunately, she subsequently presented with aggravated dry cough and dyspnea. Tumor regrowth in mediastinum lymph nodes was observed, and a diagnosis of superior vena cava obstruction syndrome (SVCS) was made. In June 2018, she was administrated with thoracic radiotherapy followed by two cycles of chemotherapy with irinotecan (120 mg/m^2^ days 1, 8, every 3 weeks) and carboplatin (area under the curve of 5 mg/ml/min, every 3 weeks) as a second-line treatment and symptoms were improved significantly. In September 2018, this patient experienced dry cough and shortness of breath again. At that time, a computed tomography (CT) scan of the chest was performed and revealed new patchy ground-grass opacities in bilateral lobes of the lung, and small right pleural effusions, without new pulmonary tumor lesions (Figure 2A). She was not found to be hypoxic. Bacterial pneumonia was excluded through negative blood and sputum culture, and progressive disease was not confirmed through fiberoptic bronchoscopy. Based on her clinical presentations and radiotherapy history, radiation-induced pneumonitis was diagnosed, and she initiated systematic steroid treatment and reported symptomatic improvement gradually (Figures 2B,C).

**Figure 3:**
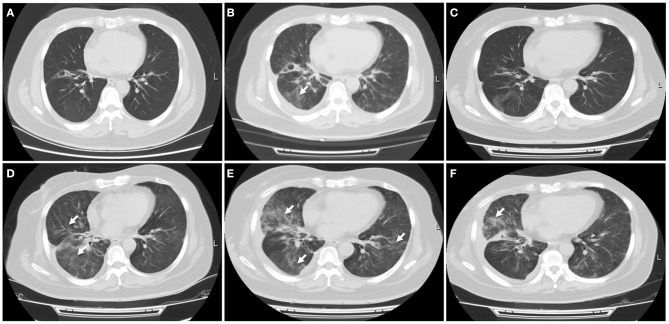
Immune-related pneumonitis. **(A)** A CT scan showed no any inflammatory lesions in the lungs following the treatment with first PD-L1 inhibitor atezolizumab. **(B)** Nivolumab was started when this patient progressed on atezolizumab treatment. A CT scan indicated new patchy ground-grass opacities in the bilateral lungs, with a small left pleural effusion. Immune-related pneumonitis was identified when blood and sputum culture did not reveal a causative microbial organism. **(C)** After treatment with corticosteroid for 1 week, this patient's symptom improved significantly, with a radiologic complete resolution of the ground-glass opacities and the pleural effusion. **(D)** Ten days later, a CT scan showed reoccurred pneumonitis, of grade 3 severity. **(E)** High-dose intravenous corticosteroid therapy did not alleviate her symptoms within 5 days, with worsening radiographic findings. **(F)** After she received immunosuppressive agents including infliximab, mycophenolate mofetil and human immunoglobulin, fever, dry cough, and dyspnea were relieved significantly, with a significant improvement of pulmonary inflammation. White arrow indicates inflammatory lesions.

In January 2019, this patient had an extensive disease progression, including multiple bone and supraclavicular lymph node metastases. Third-line nab-paclitaxel chemotherapy (200 mg days 1, 8, every 3 weeks) was started, but her tumor was not responsive to this regimen. She switched to an anti-PD-L1 antibody atezolizumab (1,200 mg every 3 weeks) therapy and local radiotherapy on lymph nodes and bone was subsequently planed and completed. In June 2019, after receiving her six cycles of immunotherapy with atezolizumab, this patient developed new liver and brain metastases. At that time a flat dose of nivolumab 240 mg every 2 weeks was started, along with further local treatment with liver and brain lesions. Just 1 week later, she experienced slight dry cough and acute new-onset fever without shortness of breath. A CT scan showed new patchy ground-grass opacities in the lung bilaterally, with a small right pleural effusion that was not present 1 month ago (Figures 3A,B). Blood and sputum culture did not reveal any causative microbial organism, and she was thought to develop immunotherapy-related pneumonitis, of grade 2 severity.

She received intravenous methylprednisolone (2 mg/kg every day for 5 days) and sequent treatment and inflammation was improved on day 15 (Figure 3C). But on day 24, she was presented with reoccurred fever, aggressive cough and dyspnea on exertion, with a low oxygen saturation of 88%. An additional CT scan showed obvious reoccurred pneumonitis of the bilateral lungs, of grade 3 severity (Figure 3D). After multidisciplinary discussion, high-dose intravenous methylprednisolone treatment (2 mg/kg every day) restarted but this did not alleviate her symptoms within 5 days, with a low oxygen saturation of 84–88%. At that time, she was firstly diagnosed with concomitant bacterial pneumonia with *Klebsiella pneumoniae*, but the use of specific antibiotics did not improve her symptoms (Figure 3E). She was continuously treated with steroid and received immunosuppressive agents including infliximab (5 mg/kg), mycophenolate mofetil (1 g twice every day), as well as intravenous immunoglobulin (IVIG; 2 g/kg every day for 5 days). After treatment with combination immunosuppression, fever, dry cough and dyspnea on exertion were relieved significantly and oxygen saturation returned to a normal level, with a significant radiographic improvement of pulmonary inflammation (Figure 3F). Unfortunately, subsequent CT scan demonstrated progressive disease in the liver.

## Discussion

Generally, both diagnosis and therapy are challengeable in identifying and managing cancer patients who may be potential PD-1/PD-L1 inhibitor-related pneumonitis. Pneumonitis can develop at any time before or after initiation of anti-PD-1/PD-L1 therapy in patients with metastatic lung cancer. Pulmonary toxicity may be a radiation recall limited to previously areas of the lung where radiation was applied. Radiation-induced lung injury including pneumonitis and fibrosis may present within several months or years following radiation therapy (13). Furthermore, unusual opportunistic infections including pneumonia can develop in patients with prolonged immune suppression which is used to treat irAEs (14, 15). Data from a single institution showed that serious infections occurred in 7.3% of advanced melanoma patients who received ipilimumab, either alone or in combination with nivolumab. The most common opportunistic infections were bacterial infection, others were viral, fungal, and parasitic (14). Thus, immune-related pneumonitis is viewed as a diagnosis of exclusion, and other completing causes for similar clinical presentation should be considered or excluded, including lung infection progressive disease in the lungs. Sometimes immune-related pneumonitis could present with concurrent infection and/or disease progression, which presents as a complication in clinical practice. In fact, preexisting pulmonary damage from inflammation, radiation, idiopathic pulmonary diseases, previous use of taxanes, gemcitabine and tyrosine kinase inhibitors, as well as increased tumor burden may increase the risk of developing immune-related pneumonitis (4, 5).

Currently, combination therapy strategies have been developed to improve PD-1 blockade efficacy in various tumor types. These include combinations with checkpoint inhibitors, radiation therapy, chemotherapy, small molecular inhibitors and several other existing cancer treatments (7, 9). It is becoming common that a second PD-1/PD-L1 inhibitor might be used following disease progression on previous PD-1/PD-L1 inhibitor. However, PD-1/PD-L1-based combination therapy leads to relatively high incidence of treatment-related adverse events. For example, the combination of osimertinib and durvalumab was associated with high incidence of interstitial lung disease (38%), leading to termination of further patient enrollment (16). Even severe irAEs also occurred frequently in endothelial growth factor receptor (EGFR)-mutant NSCLC patients who received sequential PD-1/PD-L1 inhibition and osimertinib treatment (17). In CheckMate 370 trial, 38% of anaplastic lymphoma kinase translocation (ALK)-positive NSCLC patients treated with nivolumab plus crizotinib developed severe hepatic toxicities, leading to the discontinuation of the combination and enrollment was closed earlier (18). An anti-CTLA-4 antibody in combination with an anti-PD-1 antibody increases both incidence and severity of irAEs. The overall incidence of pneumonitis for patients with anti-PD-1/PD-L1 combination therapy is 6.6% compared to 1.6% for those with monotherapy (5). These toxicities including fatal side effects also tend to be present earlier in the course of combination immunotherapy treatment and evolve rapidly compared with immune checkpoint inhibitor alone. The median time to the onset of fatal toxic event is about 14.5 days for patients with combination immune checkpoint therapy, compared to about 40 days for those treated with monotherapies (19). Although recurrent irAEs are mild and manageable, and a subgroup of patients were responsive to retreatment with previous immunotherapy, it remains unclear whether it is safe and efficacious when a patient switches to a different PD-1/PD-L1 inhibitor because of progression on the treatment with previous PD-1/PD-L1 inhibitor (Table 1) (22–24).

**Table 1:** Summary of reported cases documenting sequential treatment with different PD-1/PD-L1 inhibitors.

**Authors**	**First PD-1/PD-L1 inhibitor**	**Second PD-1/PD-L1 inhibitor**	**Regimen targets**	**Tumor type**	**Causes of switching therapy**	**Outcomes**
Martini et al. (20)	PD-L1 inhibitor	Nivolumab	PD-L1 + PD-1	RCC	PD	PD
Martini et al. (20)	PD-L1 inhibitor	Nivolumab	PD-L1 + PD-1	RCC	PD	PD
Martini et al. (20)	Pembrolizumab	Nivolumab	PD-1 + PD-1	Melanoma	PD	PD
Liu et al. (21)	Nivolumab	Atezolizumab	PD-1 + PD-L1	NSCLC	PD	Death caused by severe pneumonitis and myocarditis
Lepir et al. (22)	Nivolumab	Pembrolizumab	PD-1 + PD-1	Melanoma	PD	CR

*CR, complete response; PD, progressive disease; PD-1, programmed cell death protein-1; PD-L1, programmed cell death ligand-1; NSCLC, non-small cell lung cancer; RCC, renal cell carcinoma*.

In our case, this patient developed serious interstitial lung disease after sequential use of atezolizumab and nivolumab. To the best of our knowledge, this is the first case report involving immune-related pulmonary toxicity due to sequential therapy with different PD-1/PD-L1 inhibitors. Another similar case report mentioned that severe pneumonitis and myocarditis were identified in a patient with lung squamous cell carcinoma who received nivolumab followed by atezolizumab monotherapy treatment (21). Furthermore, although retreatment is plausible rationale and there are several ongoing trials that allow prior treatment with a PD-1/PDL1 inhibitor, there is insufficient clinical data to support the treatment with another PD-1/PD-L1 inhibitor after progression on previous PD-1 pathway blockade (20). Therefore, caution is needed in patients receiving combinational or sequential application of PD-1/PD-L1 inhibitors. Although the mechanism of action underlying sequential use of PD-L1/PD-1 inhibitor remains unknown, syngeneic tumor-bearing mice model suggested that combination of anti-PD-1 and anti-PD-L1, either sequentially or simultaneously administered, caused fulminant cardiotoxicity, and this effect is associated with infiltrating leukocyte but not CD8+ T cells accumulation in the hart. The administration of the PD-L1 inhibitor alone prior to the PD-1 inhibitor did not cause leukocytic infiltration of the myocardium (21).

Therapy and follow-up of immune-related pneumonitis remain a major challenge in the clinical practice. Treatment of pneumonitis is often determined by organizations and practice settings. For example, several guidelines or consensus for the management of irAEs in patients treated with immune checkpoint inhibitor therapy have been published (10–12). Immunotherapy teaching and monitoring tools have been developed by the National Comprehensive Cancer Network (NCCN) and can be utilized by patients and providers to monitor different irAEs related to immune checkpoint inhibitors (25). However, no prospective clinical trials have been identified that determine an optimal treatment approach for management of pneumonitis and other serious irAEs. Diagnostic evaluation and management appear to be empirical. In the majority of patients, pulmonary toxicity secondary to anti-PD-1/PD-L1 therapy can be resolved with the use of corticosteroid alone. However, a subgroup of patients cannot improve initially or completely and require additional suppressive medications because of steroid-refractory situation. In our case, this patient received high-dose corticosteroid and improve clinically after the onset of nivolumab-related pulmonary toxicity, but she rapidly developed a resistance to steroid treatment. According to published guidelines, if patients do not improve after 48 h of steroid treatment 1–2 mg/kg/d), addition of infliximab 5 mg/kg or mycophenolate mofetil intravenous 1 g twice a day or IVIG for 5 days or cyclophosphamide should be considered. Previous case report showed that single immunosuppressive medication was not insufficient (26). We considered that rapidly recurred pneumonitis was steroid refractory and used different immunosuppressive medications with infliximab and mycophenolate mofetil. In the meantime, intravenous immunoglobulin dosed at 2 g/kg was also administered. She got a good clinical response when receiving additional immunosuppressive medications. Thus, patients receiving combinational or sequential use of immune checkpoint inhibitors have a relative high risk to develop high-grade or steroid-resistant pneumonitis. Previous report showed that the addition of IVIG to high-dose corticosteroid could be viewed as an alternative therapy for steroid-refractory immune-related pneumonitis (27). Here, additional suppressive medications might be used earlier when pulmonary toxicity occurs following the combinational or sequential use of PD-1/PD-L1 inhibitors. Additionally, pulmonary infection was identified, and use of antibiotics did not produce good clinical response. However, infection screening is very important to exclude the presence of infections before considering PD-1/PD-L1-related pulmonary toxicity, regardless of a history of prior corticosteroid administration. Moreover, prospective study of early use with steroid and immunosuppressive agents in the treatment of serious immune checkpoint inhibitor-related pneumonitis is needed to establish best clinical practice in the field of immune-oncology.

## Conclusions

Combination therapy based on PD-1/PD-L1 inhibitors might be viewed as a promising therapeutic strategy for resistant lung cancer, and it is becoming common that a second PD-1/PD-L1 inhibitor might be used following the progression on previous PD-1 pathway blockade. These patients have a relative high risk to develop high-grade or steroid-resistant pneumonitis, and additional suppressive medications should be used earlier when severe pulmonary toxicity occurs. In the meantime, pulmonary infections should be excluded before considering PD-1/PD-L1-related pulmonary toxicity, to avoid a situation of misuse with corticosteroids for immune-related pneumonitis, which would be an important consideration for oncologists and immunologists.

## Data Availability Statement

The datasets generated for this study are available on request to the corresponding author.

## Ethics Statement

The studies involving human participants were reviewed and approved by The ethics committee of No. 960 Hospital of PLA. The patients/participants provided their written informed consent to participate in this study. Written informed consent was obtained from the individual(s) for the publication of any potentially identifiable images or data included in this article.

## Author Contributions

XL was involved in the identification and selection of patient cases and drafted the manuscript. YG and BZ were involved in the drafting and editing of the manuscript. BZ, YL, JL, and BW reviewed and edited the manuscript. JW was involved in the identification, selection, and management of patient cases and reviewed and edited the manuscript. All authors read and approved the final manuscript.

### Conflict of Interest

The authors declare that the research was conducted in the absence of any commercial or financial relationships that could be construed as a potential conflict of interest. The reviewer JW declared a shared affiliation, though no other collaboration, with one of the authors BZ to the handling Editor.

## Abbreviations

ALK: Anaplastic lymphoma kinase translocations
CT: Computerized tomography
CTLA-4: Cytotoxic T-lymphocyte-associated protein 4
EGFR: Endothelial growth factor receptor
FDA: United States Food and Drug Administration
irAEs: Immune-related adverse events
IVIG: Intravenous immunoglobulin
NCCN: National Comprehensive Cancer Network
NSCLC: Non-small cell lung cancer
PD-1: Programmed cell death protein-1
PD-L1: Programmed death ligand-1
SCLC: Small cell lung cancer
SVCS: Superior vena cava obstruction syndrome.
